# Multi-Omic Analysis of Cerebrospinal Fluid Metabolites in Autism Spectrum Disorder: Biomarker Identification, Metabolic Genetics Insights, and Network Toxicology

**DOI:** 10.3390/genes17080874

**Published:** 2026-07-27

**Authors:** Dan Zhao, Junzhi Guo, Ying Zhang, Yuanfeng Lan, Tian Zhao, Yiliang Xu, Qizhou Yang, Haihong Ye

**Affiliations:** 1 Department of Medical Genetics and Developmental Biology, School of Basic Medical Science, Capital Medical University, Beijing 100069, China; zhaod@mail.ccmu.edu.cn (D.Z.); gjz16666@gmail.com (J.G.); 112024040040@mail.ccmu.edu.cn (Y.Z.); yuanfeng_lan@mail.ccmu.edu.cn (Y.L.); zhaotian@ccmu.edu.cn (T.Z.); xuyl@ccmu.edu.cn (Y.X.); 2 Laboratory for Clinical Medicine, Capital Medical University, Beijing 100069, China; 3 Beijing Key Laboratory of Cell and Gene Therapy in Otology, Beijing 100069, China; 4 Department of Neurocritical Care, Beijing Anzhen Hospital, Capital Medical University, Beijing 100029, China

**Keywords:** autism spectrum disorder, cerebrospinal fluid metabolomics, environmental neurotoxicants, network toxicology, molecular docking

## Abstract

**Background**: Although genetic-environmental interactions are established in autism spectrum disorder (ASD), how environmental toxicants confer susceptibility remains unclear. This study aimed to investigate potential relationship between genetically predicted cerebrospinal fluid (CSF), metabolite levels and ASD liability, and to prioritize regulatory genes, key pathways, and candidate environmental toxicants. **Methods**: Using two ASD GWAS datasets (exploration data: 18,381 ASD cases/27,969 controls; validation data: 18,235 ASD cases/36,741 controls), we applied multi-omics approaches to prioritize ASD-associated CSF metabolites, regulatory SNPs, and genes. Enrichment analysis and protein–protein interaction (PPI) network analysis were performed on these metabolite-related genes to explore the potential mechanisms linking CSF metabolic disturbances to ASD. Finally, candidate environmental neurotoxicants were screened through protein-chemical interaction analysis, with binding relationships assessed via molecular docking prediction. **Results**: Two-sample Mendelian randomization (MR) analysis prioritized adenine and proline as candidate CSF metabolites with potential risk associations with ASD. Summary-data-based MR (SMR) prioritized 39 brain-specific quantitative trait loci (QTL) involving 35 candidate regulatory genes, including dual-metabolite modulator GRM8. Functional enrichment analyses suggested potential associations with mitochondrial dysfunction, Hippo signaling pathway, and microtubule dynamics impairment, with protein–protein interaction networks highlighting KATNA1/KATNAL2 as hubs. Protein-chemical interaction screening nominated 14 candidate environmental toxicants, including established chemicals (acetaminophen, valproic acid, estradiol) and novel candidates (SB-431542, K 7174, benzo[a]pyrene), with docking affinity assessed computationally. **Conclusions**: Our study provides suggestive evidence that elevated adenine and proline may be potential risk factors for ASD and suggests possible involvement of the mitochondrial–Hippo–microtubule pathway. We also propose benzo[a]pyrene as a candidate environmental toxicant that may perturb CSF metabolism. However, given the limited statistical significance, these findings require further validation.

## 1. Introduction

Autism spectrum disorder (ASD) is a prevalent neurodevelopmental disorder characterized by impaired social communication and restricted, repetitive behaviors [1,2]. With 70–90% heritability, ASD exhibits extreme genetic heterogeneity: over 800 rare penetrant variants and thousands of common polygenic alleles collectively drive its etiology [3,4]. Its phenotypic heterogeneity stems from complex genetic architecture involving polygenic inheritance, de novo/inherited variants (e.g., SHANK3, CHD8), epigenetic factors, and environmental influences. Key pathways disrupted include synaptic function, chromatin remodeling, and neuronal signaling. Despite advanced genomic diagnostics (WES/WGS), limited diagnostic yields reflect ASD’s etiological complexity [5,6]. Critically, comorbidities, including intellectual disability (ID), epilepsy, and attention-deficit/hyperactivity disorder (ADHD), affect approximately 70% of individuals with ASD [7,8]. Notably, environmental factors also contribute significantly to ASD etiology, with prenatal exposures to air pollutants, endocrine-disrupting chemicals, and neurotoxic compounds demonstrating particularly strong epidemiological associations [9,10]. Metabolomics provides a powerful framework for elucidating neurological pathophysiology by integrating genetic and environmental influences [11,12,13,14]. As the downstream product of genomic, transcriptomic, and proteomic processes, the metabolome captures cumulative effects of genetic predisposition and environmental exposures, including maternal immune activation, gut dysbiosis, and toxins [15,16].

Blood/urine metabolomic studies consistently identify ASD metabolic signatures featuring: (1) mitochondrial dysfunction and redox imbalance, which is evidenced by elevated levels of lactate, alanine, and acylcarnitine in blood plasma, reflecting impaired oxidative phosphorylation [17,18,19,20,21]; (2) dysregulation of one-carbon metabolism, reflected in low S-adenosylmethionine-to-S-adenosylhomocysteine (SAM/SAH) ratios in urine consistent with aberrant epigenetic regulation (including methylation abnormalities) [22,23,24,25]; (3) perturbations in neurotransmitter pathways, including the tryptophan–kynurenine, GABA–glutamate, and dopamine systems, that directly impinge on synaptic signaling [25,26,27,28]; and (4) disruption of the gut–brain axis, evidenced by atypical profiles of microbe-derived metabolites such as fatty acids, indoles, and bile acid conjugates [29,30,31,32,33].

While blood/urine metabolomic studies reveal systemic metabolic disruptions, cerebrospinal fluid (CSF) offers a direct window into the neurochemical microenvironment of the central nervous system (CNS), where core pathological processes unfold. CSF provides direct biochemical access to the CNS, where the types and concentrations of metabolites precisely mirror dynamic changes in the CNS metabolome [34,35,36]. This unique neurobiological relevance makes CSF an ideal medium for studying neurodevelopmental disorders. Despite technological advances enabling quantification of >400 metabolites from minute volumes (50 µL) [37,38], CSF metabolomics in ASD remains critically understudied. Genetic defects in key metabolic enzymes elevate specific CSF metabolites (e.g., lactate, branched-chain amino acids) and increase ASD risk [39]. Maternal auto-antibodies crossing the fetal blood-CSF barrier disrupt folate/purine metabolism and induce ASD-like behaviors in models [40,41,42]. Clinical interventions correcting metabolic anomalies (e.g., via branched-chain amino acid supplementation, folic acid, or mitochondrial cofactors) ameliorate core ASD symptoms [33,43,44,45,46]. However, current studies examining relationships between ASD and CSF metabolites remain narrowly focused on identifying individual metabolites, lacking systematic investigation of the metabolome. Therefore, systematic understanding of CSF metabolic dysregulation in ASD is very limited, particularly regarding the interaction between genetic determinants and environmental toxicants.

To enable a systematic and comprehensive analysis of the CSF metabolic environment in ASD, we integrated genetic assessment with network toxicology. This approach allowed the prioritization of metabolite-, gene-, and toxicant-level candidates potentially involved in ASD pathophysiology. Our study presented a new framework linking CSF metabolic dysregulation to ASD etiology. Alterations in ASD-prioritized candidate metabolites (adenine and proline), impaired mitochondrial metabolism, and dysregulation of the Hippo signaling pathway may collectively associate with disrupted microtubule dynamics, potentially involving KATNA1 as a hub protein. These integrated perturbations may be linked to environmental toxicants (e.g., valproic acid, acetaminophen, benzo[a]pyrene), which are candidate contributors to ASD pathogenesis through these potentially interconnected mechanisms.

## 2. Materials and Methods

### 2.1. Study Design

**Figure 1:**
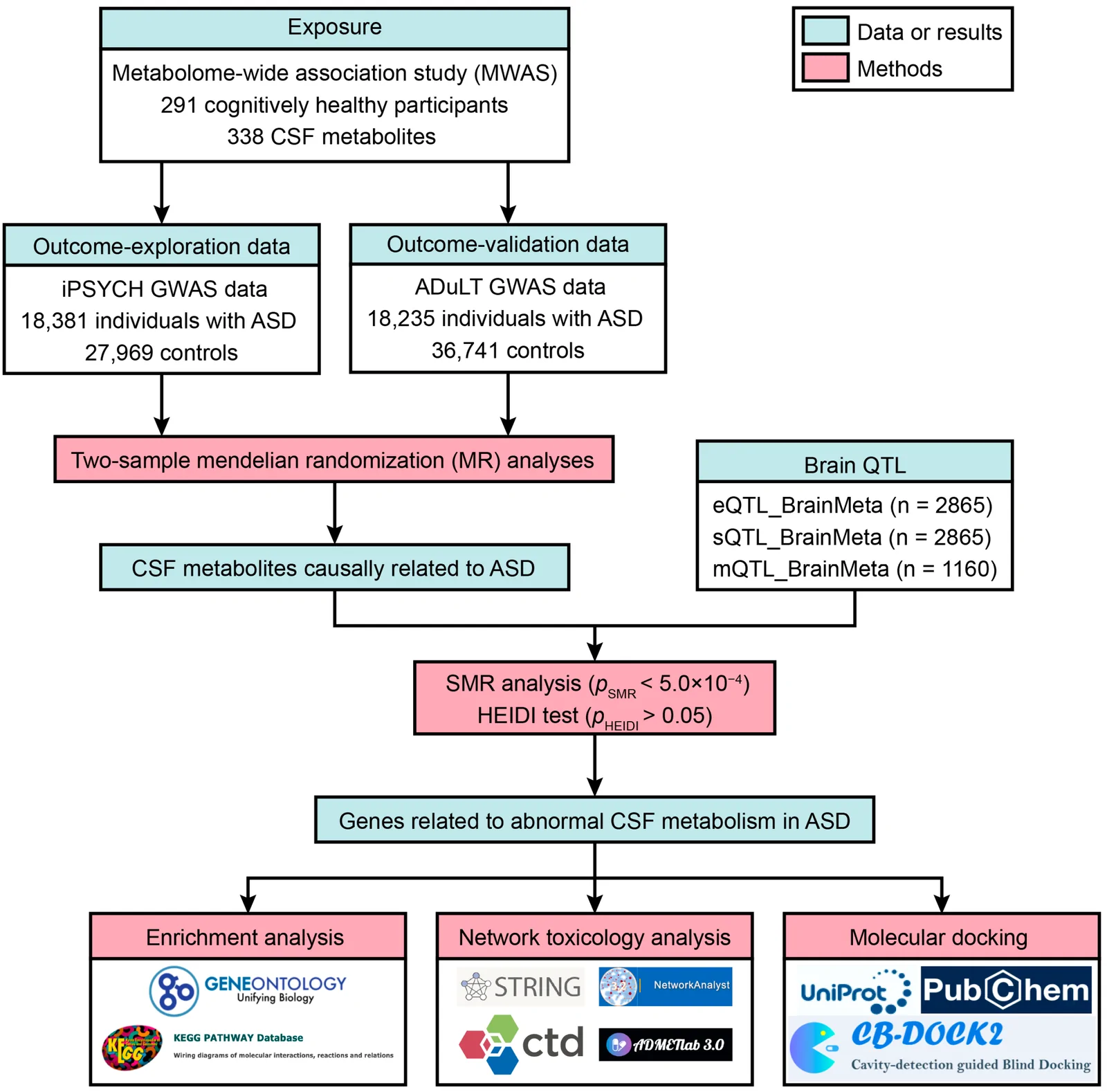
Flow chart of the study. CSF, Cerebrospinal Fluid; GWAS, genome-wide association study; QTL, quantitative trait loci; SMR, Summary-Based Mendelian Randomization; HEIDI, heterogeneity in dependent instrument; KEGG, Kyoto Encyclopedia of Genes and Genomes; GO, Gene Ontology.

In this study, we first utilized a two-sample MR analysis to nominate CSF metabolites with potential associations with ASD. Subsequently, we used SMR analysis to prioritize candidate SNP loci and their related genes that may be involved in regulating these metabolites. Enrichment analysis and protein–protein interaction (PPI) network analysis were conducted on these metabolite-associated genes to explore possible mechanisms through which CSF metabolic disturbances might contribute to ASD. Finally, screening of candidate environmental toxicants was carried out through protein-chemical interactions analysis, and their potential binding relationships were predicted using molecular docking analysis. The overall study design is illustrated in Figure 1. Details of the methods and data are provided in the following sections.

### 2.2. MR Analysis

This study utilized a metabolome-wide association study based on Wisconsin Alzheimer’s Disease Research Center (WADRC) as the exposure GWAS data [47]. The exposure GWAS data included the genetic data of 291 cognitively healthy participants of European ancestry, with a total of 338 CSF metabolites—each corresponding to an independent GWAS dataset (the specific information of each metabolite is presented in Supplementary Table S1). The outcome data were derived from large-scale cohort-based GWAS data for ASD, including two independent GWAS datasets used as exploration and validation data, respectively. The exploration data employed an ASD GWAS from the Integrative Psychiatric Research (iPSYCH) project [3], comprising 18,381 ASD cases and 27,969 controls. The validation data utilized an ASD GWAS based on the age-dependent liability threshold (ADuLT) model (18,235 ASD cases and 36,741 controls) (Figure 1) [48].

To ensure the validity of single-nucleotide polymorphisms (SNPs) as instrumental variables (IVs) in the MR analysis, rigorous quality control measures were implemented. First, SNPs associated with CSF metabolites were selected based on a genome-wide significance threshold with *p* < 5 × 10^−8^ [49]. Second, linkage disequilibrium (LD) was minimized by retaining SNPs with *R*^2^ < 0.01 within a 5 Mb window. SNPs with a minor allele frequency (MAF) of less than 0.42 were excluded to avoid strand ambiguity [50]. Additionally, *R*^2^ was calculated to measure the proportion of variance in the trait (or phenotype) that can be explained by the effect of the SNP in the model using the following equation: *R*^2^ = 2 × MAF × (1 − MAF) × β^2^, where β signifies the effect estimate of the genetic variant in the exposure GWAS. In addition, the *F*-statistic was calculated to determine the strength of the association between SNP and metabolites using the following equation: *F* = *R*^2^ × (N − 2)/(1 − *R*^2^) [51]. At least 3 SNPs and IVs with *F* > 10 were included in the next step of analysis [52].

Five primary MR methods were employed for analysis: Inverse Variance Weighted (IVW) [53], MR-Egger [54], Weighted Median (WM) [55], the simple mode, and the weighted mode [56]. A metabolite was considered associated with ASD if both of the following conditions are met: (1) at least in one method, it showed an association with ASD (nominal significance was defined as *p* < 0.05; metabolites are grouped by metabolic pathways according to the Human Metabolome Database, and Benjamini–Hochberg FDR correction was applied within each group, with an FDR-corrected *p* < 0.05 considered statistically significant); (2) the effect directions were consistent across all five methods. Subsequently, metabolites that showed significant association with ASD and consistent effect directions in both exploration and validation analyses are selected for further analysis.

To assess the robustness of the MR analysis, sensitivity analyses were conducted, which included heterogeneity tests (Cochran’s Q tests) to evaluate differences among IVs, pleiotropy detection using the MR-Egger method, and reliability assessments through the leave-one-out method. SNPs and metabolites that failed any of the three tests [the heterogeneity test (*p* < 0.05); horizontal pleiotropy test (intercept-*p* < 0.05), leave-one-out analysis (the overall effect estimate changed significantly after excluding a certain SNP)] were excluded from the final results [57]. All MR analyses were performed using the R package “TwoSampleMR” (version 0.6.20) [58,59].

### 2.3. SMR Analysis

In this study, we utilized the SMR online tool (https://yanglab.westlake.edu.cn/smr-portal/, accessed on 1 September 2025) [60] and the SMR software tool (version 1.3.2, https://yanglab.westlake.edu.cn/software/smr/#Download, accessed on 1 September 2025) [61] to perform SMR analysis and HEIDI (heterogeneity in dependent instruments) tests, aiming to evaluate the associations between brain-specific QTLs [62,63]. To identify relevant cis-QTLs, we selected those located within ±2 Mb of the target gene with a *p* < 5.0 × 10^−8^, to ensure robustly associated instruments for the SMR analysis. SNPs with MAF differences greater than 0.2 were excluded to minimize bias. To assess heterogeneity in the SMR results, the HEIDI test was used to determine whether significant differences existed between datasets. SNPs with a HEIDI *p* < 0.05 were considered to show stronger consistency between gene expression and disease association data. Default parameters in the software were applied to ensure the robustness and reproducibility of the results. Furthermore, a multi-SNP SMR analysis method (SMR-multi), combined with the HEIDI test, was employed to enhance result accuracy and exclude pleiotropy. Only results meeting the criteria (*p*_SMR_ < 5.0 × 10^−4^, *p*_SMR-multi_ < 0.05, and *p*_HEIDI_ > 0.05) were included in the final analysis. In this study, the SMR software R script “plot_SMR” (https://yanglab.westlake.edu.cn/software/smr/download/plot.zip, accessed on 1 September 2025) was used to generate SMR EffectPlots for visualizing SMR results.

### 2.4. Identification of Metabolite-Related Genes

In the SMR analysis, QTLs that were positively correlated with ASD-related CSF metabolites or negatively correlated with protective metabolites were identified as ASD-related CSF metabolic risk QTLs. In contrast, QTLs showing the opposite correlation pattern (negative with risk metabolites or positive with protective metabolites) were identified as protective QTLs. Genes associated with these risk QTLs and protective QTLs were defined as genes associated with CSF metabolism in ASD. After identifying these metabolite-related genes through SMR analysis, we queried and verified previous reports on these genes in the AutDB [64] (http://autism.mindspec.org/autdb/Welcome.do, accessed on 20 September 2025) and SFARI database (https://gene.sfari.org, accessed on 1 July 2026).

### 2.5. Function Enrichment Analysis

We performed functional enrichment analysis on genes identified in Section 2.4 to delineate the potential pathways that may influence ASD development. For analysis, *Homo sapiens* (*H*. *sapiens*) was selected as the species and a significance cut-off of *p* (or *adjusted p*) < 0.05 was applied. Gene Ontology (GO) [65] and Kyoto Encyclopedia of Genes and Genomes (KEGG) pathway [66] enrichment analyses were conducted for these associated genes using the KOBAS 3.0 web tool (http://bioinfo.org/kobas/, accessed on 10 September 2025) [67], which annotates relevant GO and KEGG terms and facilitates identifying whether specific biological processes (BP), molecular functions (MF), or cellular components (CC) are overrepresented among the identified genes. Subsequent visualization was performed via the bioinformatics online platform (https://www.bioinformatics.com.cn, accessed on 5 October 2025) to generate more intuitive graphical representations.

### 2.6. PPI Network Construction

To clarify the biological significance of these associated genes and identify hub targets regulating metabolism, we utilized the STRING 12.0 (https://string-db.org/, accessed on 1 October 2025) specifying restricted species as *H*. *sapiens* to construct a PPI network and screened for hub genes. We set a minimum interaction score threshold of 0.15 and visualized the interaction network of potential hub targets using Cytoscape (version 3.10.3) [68]. Subsequently, we computed node degrees to identify hub proteins using “Analyze Network” tool of Cytoscape.

### 2.7. Protein-Chemical Network Construction

The Comparative Toxicogenomics Database (CTD, http://ctdbase.org/, accessed on 1 February 2026) was utilized to identify chemical compounds with documented regulatory interactions on the selected hub genes identified in Section 2.6. CTD is used in conjunction with the NetworkAnalyst platform [69,70] to analyze the association between ASD hub proteins and chemicals. The resulting network was then visualized and analyzed using Cytoscape software (version 3.10.3) [68].

### 2.8. Toxicological Properties Prediction

To characterize the toxicological profiles of candidate chemicals, prediction of absorption, distribution, metabolism, and excretion (ADME) properties was conducted using the ADMETlab 3.0 (https://admetlab3.scbdd.com/, accessed on 20 February 2026) [71]. Considering that ASD is a neurodevelopmental disorder, particular attention was given to blood–brain barrier (BBB) permeability and neurotoxicity potential during the screening process.

### 2.9. Molecular Docking Prediction

The core targets were imported into the Uniprot database (https://www.uniprot.org, accessed on 20 February 2026) to retrieve their predicted 3D structures from the AlphaFold Database (https://www.alphafold.ebi.ac.uk/, accessed on 20 February 2026), while the 2D structures of toxicants were downloaded from the PubChem database (https://pubchem.ncbi.nlm.nih.gov, accessed on 20 February 2026). Protein structures and toxicant molecules were subjected to molecular docking prediction using CB-Dock2 (http://183.56.231.194:8001/cb-dock2/index.php, accessed on 1 March 2026), an integrated docking tool based on AutoDock Vina (version 1.2.0) [72]. This software calculates binding affinities for multiple docking conformations simultaneously. Docking poses with favorable binding energies (Vina score < −5.0 kcal/mol) were selected for further analysis and visualized using PyMOL (v3.1.6.1).

## 3. Results

### 3.1. Two-Sample MR Prioritizes Adenine and Proline as Candidate CSF Metabolites with Potential Relevance to ASD

**Figure 2:**
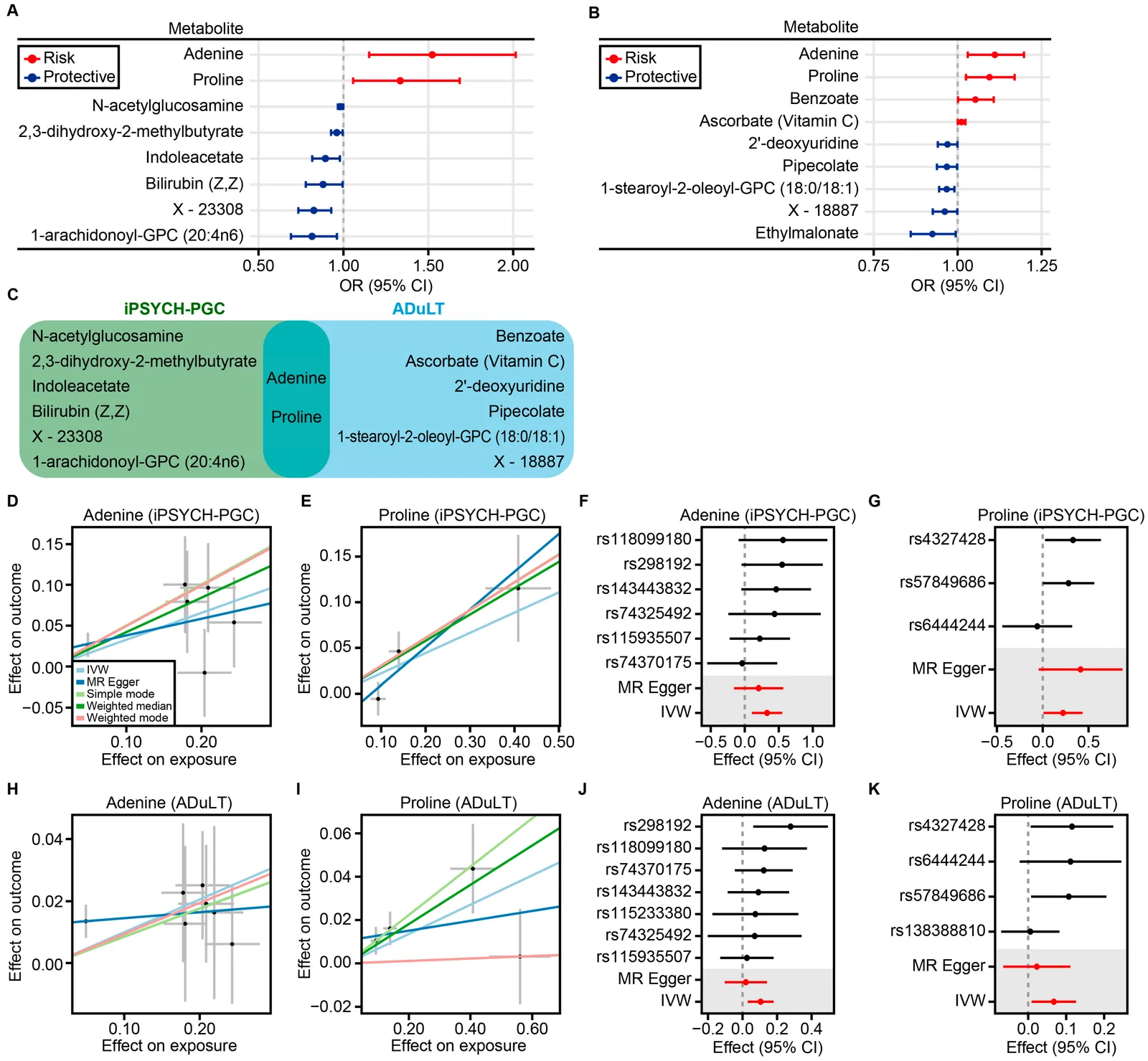
MR analysis prioritized adenine and proline as candidate metabolites with potential relevance to ASD. (**A**,**B**) Forest plots of the MR results (IVW or weighted median methods): The CSF metabolites that showed a potential relationship with ASD and were obtained by using iPSYCH-PGC (**A**, exploration data) and ADuLT (**B**, verification data) as outcome variables respectively. OR: odds ratio. CI: Confidence interval. (**C**) The Venn diagram of CSF metabolites related to ASD. The MR analysis results from both exploration and validation datasets suggested that adenine and proline may have potential risk associations with ASD. (**D**–**K**) Scatter plot of adenine (**D**,**H**) and proline (**E**,**I**) on ASD (**D**,**E** are based on iPSYCH-PGC; **H**,**I** are based on ADuLT), showing the effect estimates of SNPs on outcome variables under various MR test methods. Each point in the figure represents a selected SNP. The horizontal axis for each point denotes the effect of the IV on the exposure, while the vertical axis denotes its effect on the outcome. The slope of the regression line indicates the overall effect of the exposure on the outcome. The MR Forest plots showed the MR results of the association between multiple single SNP loci corresponding to adenine (**F**,**J**), proline (**G**,**K**) and ASD (**F**,**G** are based on iPSYCH-PGC; **G**,**K** are based on ADuLT). The horizontal axis represents the individual (the black lines) and combined effects (the red lines) of SNPS on ASD (the outcome), while the vertical axis represents each individual SNP and the combined effect estimates derived from the two MR methods: IVW and MR-Egger.

Comprehensive two-sample Mendelian randomization analyses prioritized eight candidate CSF metabolites in the exploration cohort and nine in the validation cohort with potential relevance to ASD (*p* < 0.05) (Figure 2A,B; Supplementary Tables S2 and S3). Among these, adenine and proline overlapped between the two cohorts (significantly associated with ASD, FDR-adjusted *p* < 0.05), resulting in a total of 15 unique metabolites that may warrant further investigation.

Next, to assess the robustness of these potential links, we conducted IVs’ sensitivity analyses on these 15 metabolites using three approaches: Cochran’s Q test (heterogeneity test), horizontal pleiotropy test, and leave-one-out analysis (Supplementary Tables S4–S6). Notably, in the Cochran’s Q test, ethylmalonate showed significant heterogeneity (Q-*p* = 0.02, Supplementary Table S4), raising the possibility that not all IV-derived effect estimates were consistent for this metabolite. The remaining 14 metabolites exhibited consistent effects across IVs (Q-*p* > 0.05), suggesting unbiased and homogeneous effects estimated by all included IVs. In the horizontal pleiotropy tests, no significant horizontal pleiotropy (*p* < 0.05) was detected for any of these 15 metabolites, indicating that IVs primarily influence ASD through metabolite—consistent with MR core assumptions (Supplementary Table S4). Leave-one-out analysis on the 15 metabolites further suggested that no single SNP appeared to disproportionately drive the estimates, as exclusion of individual IVs did not substantially alter results (Supplementary Tables S5 and S6, Supplementary Figures S1 and S2). Collectively, these sensitivity analyses provide suggestive support for potential links between 14 CSF metabolites and ASD, with ethylmalonate excluded due to heterogeneity concerns.

Among the eight CSF metabolites associated with ASD in the exploration cohort, 2 functioned as risk metabolites [odds ratios (ORs) > 1; 95% confidence intervals (CIs) > 1], while six showed protective effects (ORs < 1; 95% CIs < 1; Figure 2A). The validation cohort revealed four risk and five protective metabolites among its nine candidates (Figure 2B). Table 1 details these 14 metabolites (excluding ethylmalonate), annotated using the Human Metabolome Database (HMDB, https://hmdb.ca, accessed on 1 September 2025).

Notably, adenine and proline emerged as shared risk-direction candidates across both cohorts (Figure 2C). Their potential associations with ASD exhibited consistent effect directions across all five MR methods (exploration cohort: Figure 2D,E; validation cohort: Figure 2H,I). Forest plots further suggested general IV stability, showing individual SNP effect estimates for adenine and proline on ASD, respectively (exploration cohort: Figure 2F,G; validation cohort: Figure 2J,K). After metabolite pathway-specific stratified FDR correction, adenine and proline remained significant in both cohorts (Supplementary Tables S2 and S3). These two metabolites were therefore prioritized as candidate CSF metabolites for further exploratory analyses. Collectively, these findings indicate that adenine and proline show consistent suggestive associations with ASD, highlighting their potential role in ASD pathogenesis and warranting further investigation.

### 3.2. SMR Reveals QTLs and Genes with Potential Regulatory Links to Adenine/Proline Levels in CSF

**Figure 3:**
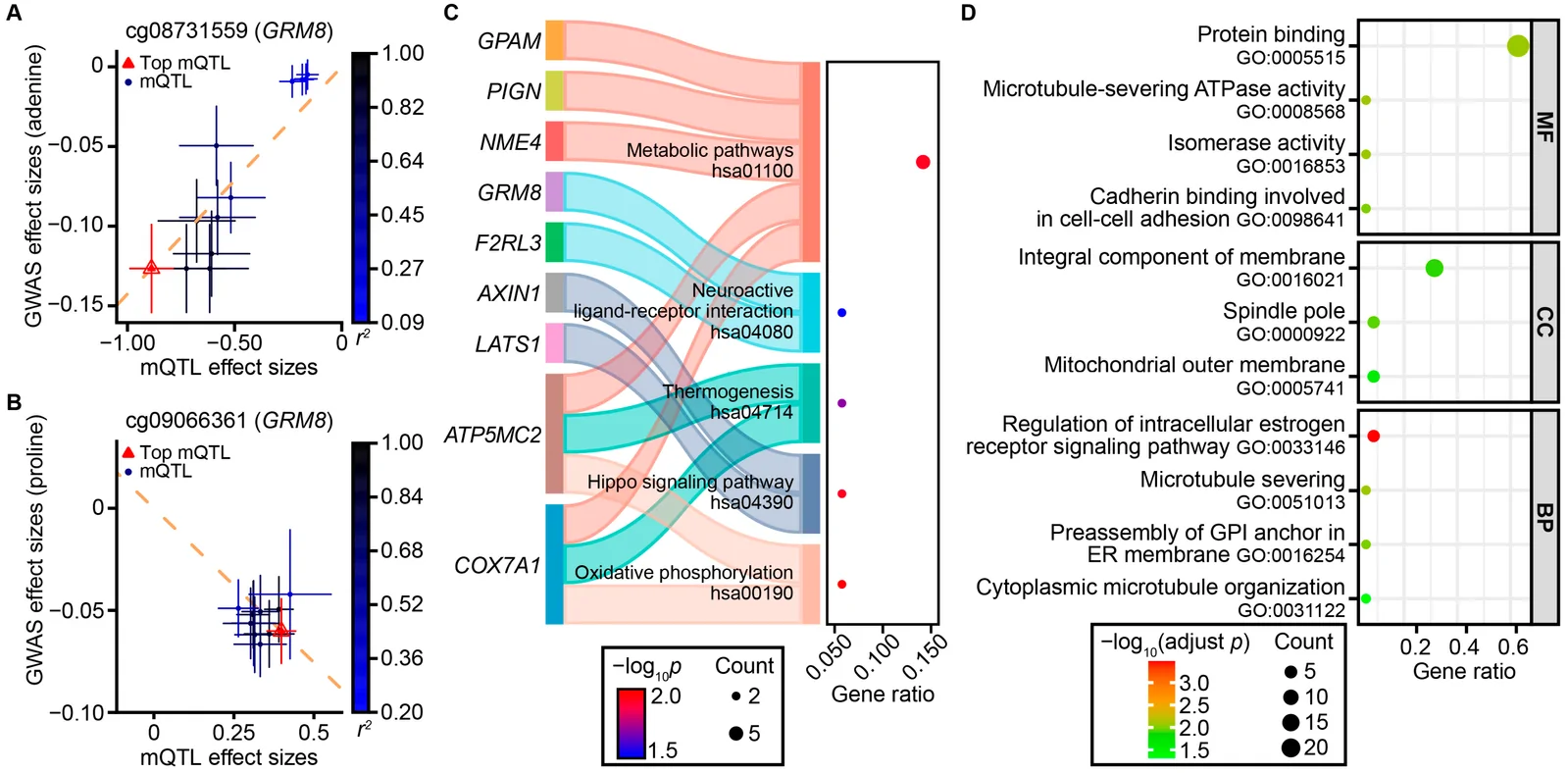
SMR EffectPlots of GRM8 and gene functional enrichment. (**A**,**B**) SMR EffectPlot comparing the effect sizes of SNPs in adenine-GWAS (**A**) and proline-GWAS (**B**) with SNPs in Brain-mMeta mQTL summary data of GRM8. Effect sizes of SNPs from GWAS (y-axis) are plotted against corresponding effect sizes from mQTL (x-axis). Each point represents an individual SNP, with color intensity scaled to LD *r*^2^ values, signifying that SNPs with high *r*^2^ serve as robust IVs for QTL-metabolite associations. Orange dashed lines: top QTL effect estimate (Beta-metabolite/Beta-mQTL). Error bars: standard errors. (**C**) Integrated Sankey-Dot plot of KEGG pathway enrichment. Left (Sankey plot): gene-pathway connections. Right (Dot): enriched pathways; dot size = gene count, color = *p* value. (**D**) GO enrichment dot plot. X-axis: gene ratio; Y-axis: pathways. Dot size = gene count, color = *p* value.

To further explore potential candidate ASD-related genes that may affect CSF metabolism, we performed Summary-Based Mendelian Randomization (SMR) analyses for adenine and proline by integrating their metabolite GWAS data with BrainMeta-QTL summary data (brain-specific eQTLs, sQTLs, and mQTLs). We then applied the HEIDI test to distinguish pleiotropy (shared related variant between GWAS and QTL signals) from linkage (distinct related variants driving signals at the same locus). QTLs meeting stringent criteria (*p*_SMR_ < 5.0 × 10^−4^, *p*_SMR-multi_ < 0.05, *p*_HEIDI_ > 0.05) were considered as potentially prioritized through colocalization with metabolite signals. These analyses prioritized a total of 39 QTLs in 35 genes: 21 QTLs in 19 genes associated with adenine (Table 2, detail results in Supplementary Table S7) and 18 QTLs in 17 genes with potential links to proline (Table 3, detail results in Supplementary Table S8). Among all genes with prioritized colocalization evidence for adenine and proline levels, four genes exhibited established ASD associations in AutDB (a curated ASD genetic database): *BAIAP2L1*, *LRP2BP*, *KATNAL2*, and *GRM8* (Supplementary Figure S3A–C). Notably, one gene, *GRM8*, shows suggestive colocalization signals for both adenine and proline levels (Figure 3A,B). *GRM8* encodes glutamate metabotropic receptor 8 (mGluR8), a G protein-coupled receptor that inhibits adenylate cyclase to reduce cAMP (cyclic Adenosine Monophosphate) synthesis upon glutamate binding [73,74].

To functionally characterize the 35 genes, we conducted Gene Ontology (GO) and KEGG pathway enrichment analyses. KEGG analysis revealed several significantly enriched pathways, including neuroactive ligand–receptor interactions (hsa04080) governing neural communication, the Hippo signaling (hsa04390) maintaining cellular growth regulation and homeostasis, and oxidative phosphorylation (hsa00190) (Figure 3C). Notably, *COX7A1* and *ATP5MC2* (Supplementary Figure S3D,E) emerged as key genes within the oxidative phosphorylation pathway (hsa00190; Figure 3C). *COX7A1* encodes a structural subunit of Complex IV (cytochrome c oxidase) in the electron transport chain, whereas *ATP5MC2* encodes a component of the c-ring subunit of mitochondrial ATP synthase (Complex V). Both proteins play essential roles in oxidative phosphorylation by facilitating proton gradient-driven ATP synthesis. The Hippo signaling (hsa04390; Figure 3C) is a highly conserved signaling cascade that integrates diverse molecular signals and biophysical states, including cell polarity, adhesion, GPCR signaling, and cytoskeleton-mediated mechanical forces [89]. The proline-related gene *AXIN1* (Supplementary Figure S3F) functions as a scaffold protein-facilitating assembly of the core Hippo signaling module (MST1/2-SAV1-WWC1-3). The adenine-related gene *LATS1* (Supplementary Figure S3G) encodes Large Tumor Suppressor Kinase 1 (LATS1), a core Hippo pathway component that, together with LATS2, forms the pathway’s gatekeeper kinases. Together, these findings suggest that Hippo signaling modulates CSF metabolic dynamics in ASD.

GO analysis revealed enrichment across three domains: molecular functions as in microtubule-severing ATPase activity (GO:0008568); cellular components as in mitochondrial outer membrane (GO:0005741) and spindle pole (GO:0000922); biological processes as in estrogen receptor signaling (GO:0033146); cytoskeletal microtubule organization (GO:0031122) and microtubule severing (GO:0051013) (Figure 3D). Collectively, these results indicate that genes regulating adenine and proline levels are functionally linked to neural signaling pathways and mitochondrial functions. GO enrichment analysis revealed multiple microtubule-severing-related processes. Given its established roles in mitochondrial energy metabolism and neural signal transmission [90,91,92], microtubule severing represents a plausible pathogenic mechanism in ASD.

### 3.3. Integrated Network Analysis Prioritized Candidate ASD-Associated Hub Proteins and Environmental Toxicants

**Figure 4:**
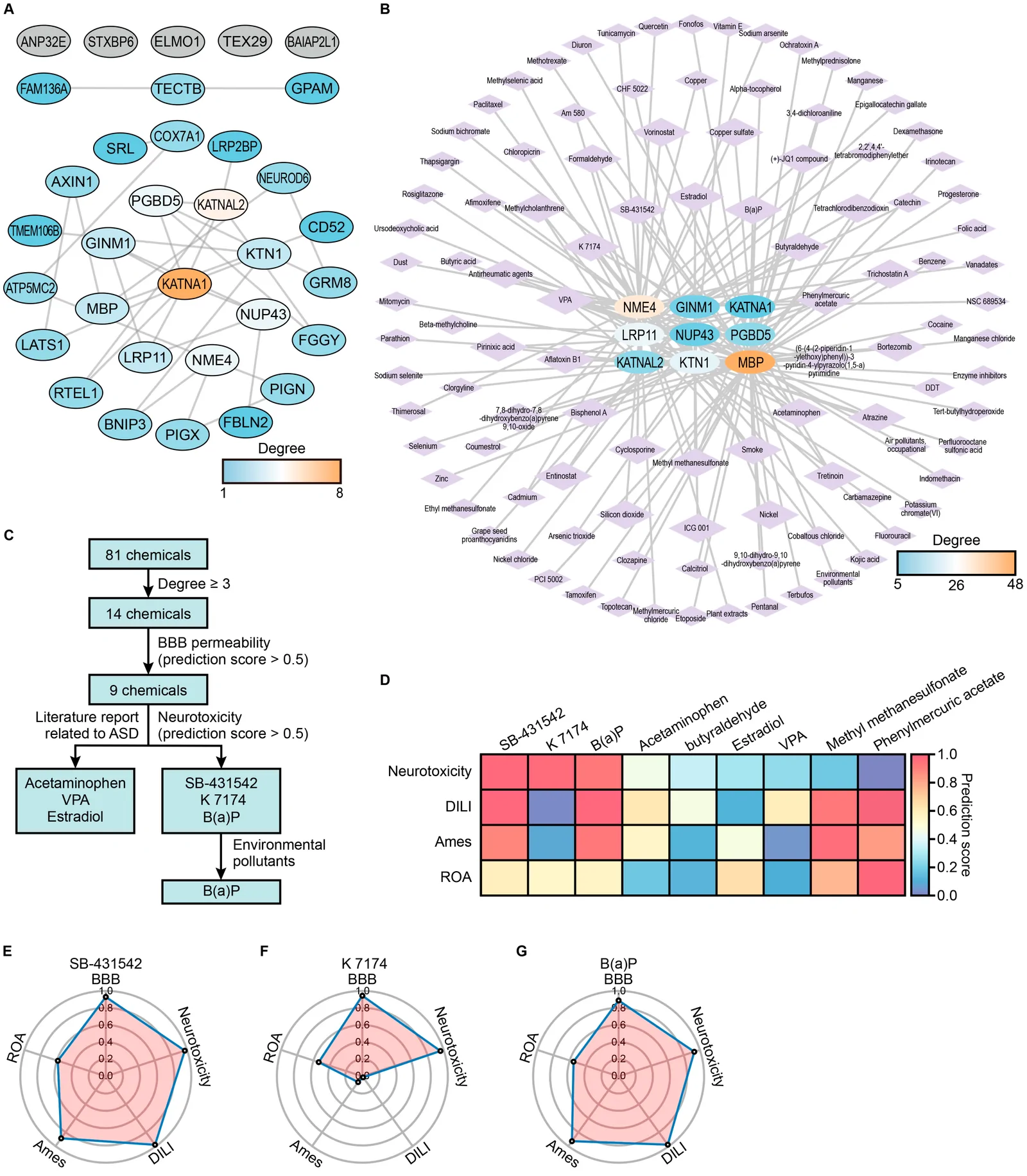
Network toxicology analysis and toxicological properties prediction. (**A**) Integrated PPI network conducted by STRING and Cytoscape. Combined PPI network of adenine/proline-associated proteins. Nodes: metabolite-associated targets; color gradient (orange intensity) indicates node degree (darker = higher degree). Edges: protein interactions. (**B**) Protein-chemical network based on CTD. Elliptical nodes: core targets (degree > 2 in (**A**)). Rectangular nodes: environmental chemicals. Edges: protein-chemical interactions. (**C**) The toxicant screening flowchart integrated prediction scores from ADMETlab 3.0 (https://admetlab3.scbdd.com/, accessed on 20 February 2026). (**D**) Toxicity prediction heatmap for 9 blood–brain barrier (BBB)-penetrant chemicals. (**E**–**G**) The toxicological properties prediction radar plots of SB-431542 (**E**), K 7174 (**F**), and B(a)P (**G**). BBB: Blood–brain barrier penetration, the output value is the probability of being BBB+, within the range of 0 to 1 (Category 1: BBB+; category 0: BBB−). Neurotoxicity: Drug-induced neurotoxicity, the output value is the probability of being neurotoxic+). ROA: Rat Oral Acute Toxicity (Category 1: high toxicity, <500 mg/kg; Category 0: low toxicity, >500 mg/kg). Ames: Ames (Bacterial Reverse Mutation Test) Toxicity (Category 1: Ames positive+; Category 0: Ames negative−). DILI: Drug induced liver injury, the output value is the probability of being toxic (Category 1: drug with a high risk of DILI; Category 0: drug with no risk of DILI).

To identify core proteins, we constructed protein–protein interaction (PPI) networks for the proteins encoded by the 35 adenine- and proline-associated genes using the STRING database (Figure 4A; protein names of 35 genes are listed in Supplementary Table S9, detailed information of PPI network is in Supplementary Table S10). The resulting integrated network consisted of 25 nodes and 35 edges, with KATNA1 (degree = 8) and KATNAL2 (degree = 5) identified as the top two hub proteins (Figure 4A; Supplementary Table S10). Both KATNA1 and KATNAL2 are components of the katanin protein complex, where KATNA1 maintains microtubule dynamics (GO:0008568, microtubule-severing ATPase activity) and KATNAL2 has been implicated in ASD pathogenesis [85], suggesting a link between microtubule dynamics and ASD.

Other highly connected hub proteins (degree ≥ 3) in the PPI network pointed to additional potential pathological mechanisms. MBP (degree = 3) is a major structural protein of the central nervous system myelin, and its deficiency may contribute to neurodevelopmental abnormalities through compromised myelin integrity [93]. NME4 (degree = 4) is a mitochondrial nucleoside diphosphate kinase that affects neuronal energy supply including mitochondrial lipid metabolism by regulating acetyl-CoA and malonyl-CoA levels [94]. LRP11 (degree = 3) participates in cholesterol homeostasis [95], thereby indirectly impacting neurodevelopmental processes.

To identify environmental toxicants potentially linked to ASD pathogenesis, we next constructed a protein-chemical interaction network using NetworkAnalyst. The analysis was based on 9 hub proteins (degree ≥ 3; KATNA1, KATNAL2, KTN1, GINM1, MBP, LRP11, PGBD5, NUP43, NME4) from our integrated PPI network (Figure 4A), with chemical information sourced from the Comparative Toxicogenomics Database (CTD). The resulting network consisted of 90 nodes (81 chemicals and 9 proteins) and 163 edges (Figure 4B; Supplementary Table S11). Among them, 14 hub chemicals (degree ≥ 3) were prioritized for further toxicological property prediction (Figure 4C).

Nine hub chemicals exhibited BBB permeability (ADMETlab prediction score > 0.5; Figure 4C, Supplementary Table S12). Three of them, acetaminophen (Supplementary Figure S4A), valproic acid (VPA) (Supplementary Figure S4D), and estradiol (Supplementary Figure S4C), are already epidemiologically associated with ASD [96,97,98,99,100,101,102,103,104] and exhibited high BBB permeability. Neurotoxicity screening (ADMETlab neurotoxicity prediction score > 0.5, Figure 4C,D) identified 3 additional candidate environmental toxicants: 4-(5-benzo(1,3)dioxol-5-yl-4-pyridin-2-yl-1H-imidazol-2-yl)benzamide (SB-431542) (Figure 4E), K 7174 (Figure 4F), and benzo(a)pyrene [B(a)P] (Figure 4G). It is notably that B(a)P, as an air and food pollutant, not only displayed high neurotoxicity but also scored elevated across multiple toxicity dimensions [including rat oral acute toxicity (ROA), bacterial reverse mutation test (Ames) toxicity, and drug-induced liver injury (DILI)], emerging as a prioritized toxicant candidate for future functional investigation.

### 3.4. Molecular Docking Prediction Revealed the Stable Binding Pattern of B(a)P

**Figure 5:**
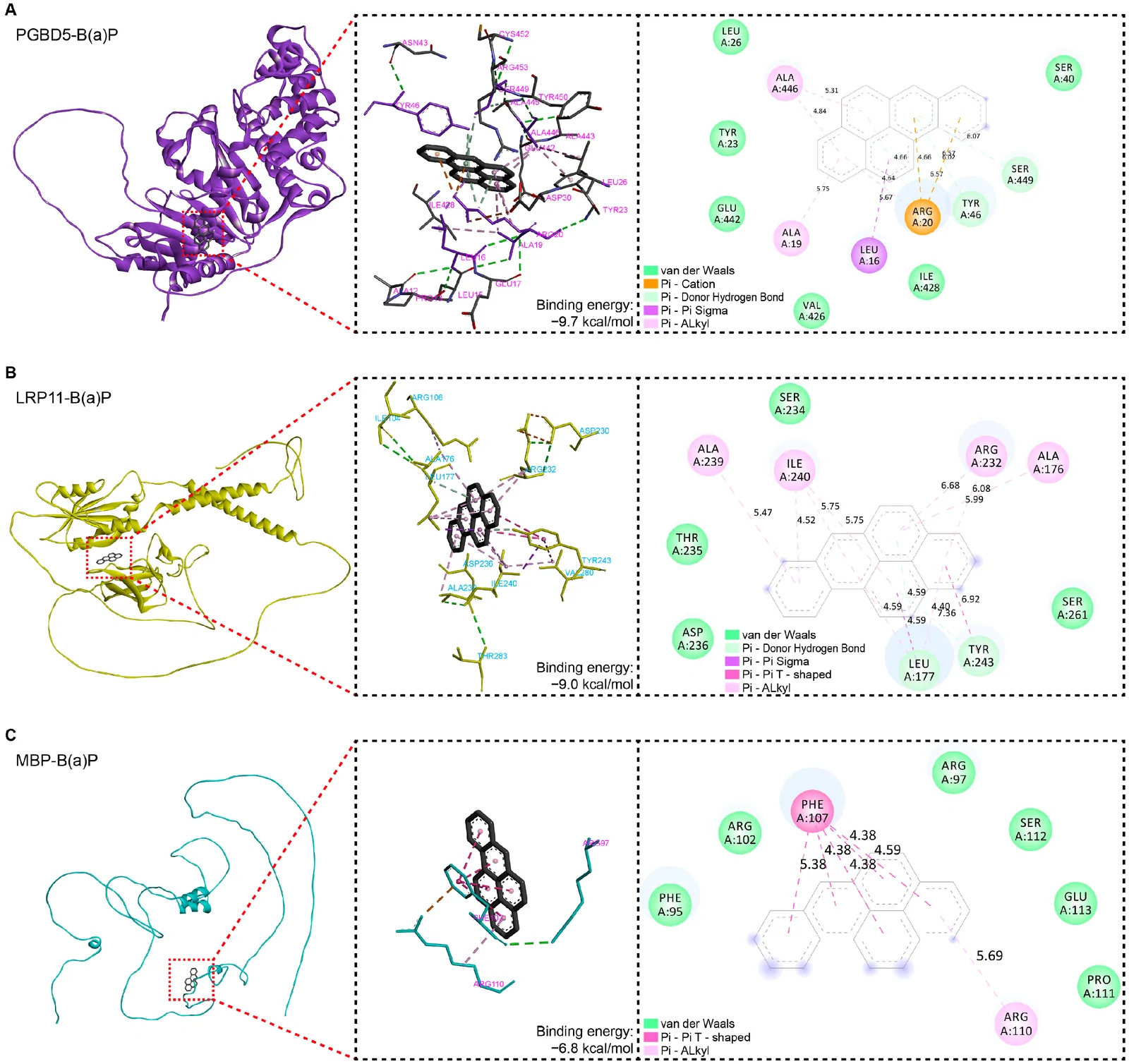
Molecular docking prediction of B(a)P binding to core target proteins. (**A**–**C**) 3D visualization and 2D interaction diagram of predicted binding poses for B(a)P with each target. The stick structures are amino acid residues. The B(a)P ligand is represented as black sticks. Different colored lines and residue circles correspond to distinct interaction types, including van der Waals forces, Pi-Alkyl, Pi-Sigma, Pi-Pi T-shaped stacking and Pi-donor hydrogen bonds. The numbers on the dashed lines represent the distance from atom to π ring measured in angstroms (Å). Binding energy refers to the binding free energy calculated by the CB-DOCK2 tool during molecular docking. The larger the absolute value, the more stable the binding. (**A**) PGBD5-B(a)P; (**B**) LRP11-B(a)P; (**C**) MBP-B(a)P.

To further reveal the interaction relationship between ASD-associated hub proteins and prioritized environmental toxicants, molecular docking prediction was used to simulate their binding patterns. The calculated binding energy values for all toxicant-protein pairs are summarized in Supplementary Table S13, providing a systematic assessment of interaction strengths. Notably, most pairs showed relatively high binding affinity to the target protein (binding energy < −5.0 kcal/mol). Especially our selected important neurotoxic environmental pollutant B(a)P exhibited high binding energies to the target protein. The binding conformations and the key amino acids involved in the binding are illustrated in Figure 5. The B(a)P binding highlighted significant interactions with residues such as ARG-20, LEU-16, ARG-232, ILE-240, and PHE-107, further supported by additional intermolecular forces (such as van der Waals forces, hydrophobic forces, Pi-donor hydrogen bonds), forming a more stable binding conformation. Based on molecular docking analyses, B(a)P showed potential to interact with target proteins, exhibiting a stronger predicted binding affinity. This evidence positions B(a)P as a high-priority candidate for future functional investigation aimed at verifying its binding and evaluating its specific mechanisms as an environmental factor in the pathogenesis of ASD.

## 4. Discussion

The escalating global incidence of ASD underscores the urgent need for early identification of environmental risk factors and development of targeted preventive interventions [105,106]. Our study identified two candidate CSF metabolites, adenine and proline, and their related genes using GWAS data, subsequently mapping these to core pathogenic proteins via PPI network analysis. Crucially, network toxicology linked these hub proteins to three prioritized environmental toxicants: SB-431542, K 7174 and benzo[a]pyrene. These findings may provide a theoretical basis for precision medicine interventions targeting gene–environment interactions, but require experimental validation before clinical translation. This foundation could facilitate the development of targeted ASD treatment regimens and contribute to the paradigm shift from traditional symptom management toward precision medicine approaches.

### 4.1. Possible Pathological Mechanisms and Biomarker Potential of Candidate Metabolites in ASD: Adenine/Proline

Using two-sample MR analyses, we identified metabolite signatures potentially associated with ASD. Adenine and proline emerged as high-priority candidates showing consistent associations across both exploration and validation GWAS datasets (Figure 2; Supplementary Tables S2–S6). Given that each pathway represents an independent biological module with distinct regulatory mechanisms, the FDR correction was applied separately within each pathway to control the false discovery rate for that specific biological process, rather than across all unrelated metabolites, to balance discovery power with biological interpretability [107]. We grouped metabolites by metabolic pathways and performed FDR correction within groups, finding that the association between adenine, proline and ASD remained significant, further highlighting their biomarker potential in ASD. Aberrant adenine metabolism disrupts nucleotide synthesis during purine metabolism, compromising essential cellular processes, including genetic integrity and energy metabolism [108]. Furthermore, adenine impairs mitochondrial function, generating oxidative stress and energy deficits that align with established ASD pathophysiology [17,18,19,21,28]. Proline is a glutamate-derived amino acid functioned as an endogenous excitotoxin. Intracerebral administration of proline in rats induces non-selective degeneration of pyramidal and granule cells [109,110], confirming its neurotoxic potential.

A notable methodological limitation is that, although we maintained a strict genome-wide significance threshold and strength for IVs selection (*p* < 5 × 10^−8^, *F* statistic > 10), the number of IVs obtained for subsequent analysis remains limited. This result was necessitated by the constrained sample size of the exposure GWAS (CSF metabolites) and the limited availability of genetic variants meeting strict *F*-statistic thresholds. GWAS data originate from the WADRC cohort (non-ASD), selected as the largest harmonized CSF metabolome GWAS for optimal QTL detection power. Because the exposure data were generated in cognitively healthy adults rather than ASD-specific cohorts, the present findings should be interpreted as cross-cohort prioritization signals requiring validation in disease-relevant populations. While sensitivity analyses mitigate concerns, residual weak instrument bias persists [111]. Future studies should prioritize larger metabolite GWAS with stronger instruments for validation.

### 4.2. Integrated Multi-Omics Revealed a Potential Mitochondrial-Hippo-Microtubule Axis in ASD Pathogenesis

Integration of multi-omic quantitative trait loci—including gene expression (eQTL), DNA methylation (mQTL), and alternative splicing (sQTL) data—through SMR analysis, prioritized 39 QTL sites involving 35 genes with suggestive colocalization evidence related to ASD (Table 2 and Table 3). Four genes [*BAIAP2L1* [75,76,77,78], *LRP2BP* [87,88], *KATNAL2* [83,84,85,86], and *GRM8* [79,80,81,82]], have been previously implicated in ASD, which supports the plausibility of our prioritization. *KATNAL2* (MIM: 614697; SFARI score 1: High Confidence) and *BAIAP2L1* (MIM: 611877; SFARI score 3: suggestive evidence) demonstrate established ASD associations in the SFARI database [76,77,78,79,84,85,86,87]. *LRP2BP* (MIM: 619020) and *GRM8* (MIM: 601116), although not mentioned in the SFARI gene database, have been reported in multiple studies to have genetic associations with ASD and are identified as candidate genes [80,81,82,83,88,89]. Notably, *GRM8* emerged as the sole gene showing suggestive colocalization signals for both adenine and proline (Figure 3A,B). Mechanistically, *GRM8* encodes metabotropic glutamate receptor 8 (mGluR8), a G protein-coupled receptor that inhibits adenylate cyclase activity, reducing cAMP synthesis [79]. This cAMP-mediated pathway potentially coordinates adenine and proline metabolic regulation. However, it is important to emphasize that SMR analysis is an indirect inference approach that relies on assumptions regarding pleiotropy, local LD structure, and the cellular/molecular context of the QTL data employed. Consequently, the prioritized genes should be regarded as statistically supported candidate regulators for further functional investigation, rather than as experimentally validated direct regulators of adenine/proline metabolism in ASD.

GO and KEGG enrichment analyses revealed significant enrichment of the 35 genes in oxidative phosphorylation (hsa00190; Figure 3C), mitochondrial outer membrane (GO:0005741; Figure 3D), Hippo signaling pathway (hsa04390; Figure 3C) and microtubule-severing ATPase activity (GO:0008568). This convergence suggests that these genes may contribute to dual neurobiological functions in neural signal transmission and mitochondrial energy metabolism, potentially coordinating through microtubule-severing mechanisms.

The enrichment of mitochondrial metabolism aligns with reported energy metabolism abnormalities in ASD pathology. Clinical studies confirm frequent mitochondrial dysfunction and metabolite alterations in ASD patients [112,113]. As cellular powerhouses, mitochondria generate ATP to fuel biological processes, and their impairment could disrupt energy metabolism and compromise cellular function. While mitochondrial dysfunction is recognized in ASD (involving metabolic dysregulation, genetic variants, and cerebral energy deficits) [114,115,116,117], the mechanistic roles of CSF metabolite-associated genes, like *COX7A1* and *ATP5MC2* (Supplementary Figure S3D,E), remain uncharacterized. *COX7A1* encodes a Complex IV subunit essential for mitochondrial respiratory chain electron transport [118,119]. *ATP5MC2* produces a c-ring component of ATP synthase (Complex V) [120]. Both components facilitate proton gradient-driven ATP synthesis during oxidative phosphorylation. Genetic variations in these genes might impair respiratory chain function and disrupt energy homeostasis, potentially contributing to ASD development.

Notably, our analyses suggest Hippo signaling may influence CSF metabolic dynamics in ASD. The Hippo pathway (hsa04390; Figure 3C) is a highly conserved signaling cascade integrating diverse molecular signals and biophysical cues—including cell polarity, adhesion, GPCR signaling, and cytoskeleton-mediated mechanical forces [89]. Recent studies establish its essential roles throughout neurodevelopment: from embryogenesis (implantation/gastrulation) to neuronal differentiation [121]. Hippo signaling interacts with key developmental pathways, including Wnt, Notch, and TGF-β [122,123,124], where Wnt pathway dysregulation impacts neuronal fate specification and dendritic morphogenesis [125,126,127,128,129,130,131]. Our enrichment analysis identified Hippo pathway components AXIN1 (proline-associated; Supplementary Figure S3F) and LATS1 (adenine-associated; Supplementary Figure S3G). According to established literature, AXIN1 scaffolds the core Hippo module [123], while LATS1 phosphorylates YAP/TAZ to regulate nuclear translocation and transcriptional programs controlling cell proliferation/apoptosis [132]. These literature-established roles suggest potential Hippo-mediated mechanisms in ASD pathogenesis.

Our PPI network analysis prioritized KATNA1 (degree = 8) and KATNAL2 (degree = 5) as the top hub proteins (Figure 4A; Supplementary Table S10). Both belong to the katanin complex—a critical regulator of microtubule dynamics. While KATNAL2 is an established ASD-risk gene whose loss disrupts microtubule dynamics and ciliary function [85]. KATNA1 maintains microtubule homeostasis through transcriptional regulation of severing genes (such as itself and SPAST, FIGN, etc.) [133]. Previous studies show microtubule severing affects mitochondrial transport, functional localization, and energy metabolism by regulating microtubule network dynamics, and also participates in synapse pruning, axon regeneration, and signal transmission by reshaping the microtubule cytoskeleton in neurons [90,91,92]. These processes collectively form a coordinated regulatory network of mitochondrial–Hippo–microtubule axis. Based on our network topology findings, KATNA1’s hub status positions it as a candidate factor warranting systematic investigation in ASD. While our STRING analysis employed a permissive confidence threshold (0.15) without evidence filtering to maximize network sensitivity, this approach may have included biologically weak interactions. Future research should conduct more rigorous experiments or apply stricter selection criteria to verify these interactions.

### 4.3. Environmental Considerations of ASD: Prioritized Toxicant Candidates for Future Investigation

Expanding to environmental interactions, we integrated toxicant exposure and endogenous metabolic regulation to screen potential ASD-risk chemicals (Figure 4B–G Supplementary Tables S11 and S12). Multiple epidemiological studies have shown that exposure to acetaminophen [103,104,134] and VPA [96,97,100,101,102] during pregnancy significantly increases the risk of neurodevelopmental disorders in offspring, including ASD. Low levels of estradiol are associated with a higher risk of ASD accompanied by intellectual disability (ID) [135]. Beyond confirming previously reported ASD-associated chemicals, our study prioritized three candidate environmental toxicants for ASD—SB-431542 (TGF-β type I receptor kinase activity inhibitor), K 7174 (cell adhesion modulator), and B(a)P (ubiquitous air pollutant)—extending beyond conventional genetic frameworks (Figure 4E–G). SB-431542 significantly promotes cell proliferation by inhibiting TGF-β signaling, but simultaneously inhibits the migratory ability of neural stem cells [136]. This dual effect suggests it may disrupt the cellular dynamic balance during neurodevelopment. Among these candidates, B(a)P emerged as an environmental toxicant candidate for future functional investigation. Our molecular docking predictions suggest a potential for stable binding of B(a)P to the target proteins (binding energy < −5.0 kcal/mol). B(a)P exposure may influence neural function through effects on neural stem cell dynamics [137] or epigenetic modifications, including circRNA alterations [138]. Our computational prioritization highlights B(a)P as a candidate toxicant that may warrant investigation for potential gene–environment interactions in ASD. These observations may inform future mechanistic and environmental studies, but do not establish a direct preventive implication. Future work examining relationships between such prioritized toxicants and ASD-susceptibility genes could help identify critical developmental windows that may be relevant to risk assessment.

## 5. Conclusions

Our integrated multi-omics approach provided suggestive evidence for candidate ASD-associated CSF metabolites, genes, and environmental toxicants. The analyses are consistent with the potential involvement of mitochondrial dysfunction and raise the possibility of a mitochondrial–Hippo–microtubule axis that may contribute to ASD pathogenesis. Network toxicology and molecular docking prediction proposed B(a)P as a candidate environmental neurotoxicant for future investigation. However, given the limited statistical significance, these findings require further validation. These metabolite–gene–environment interactions could inform precision medicine approaches for early detection and may represent a hypothesis warranting future study in prevention-oriented research.

## Figures and Tables

**Table 1:** ASD-related CSF metabolites.

Biochemical Name	Effect Direction	Super Pathway	Sub Pathway	KEGG	HMDB
adenine	risk	Nucleotide	Purine Metabolism, Adenine containing	C00147	HMDB00034
proline	risk	Amino Acid	Urea cycle; Arginine and Proline Metabolism	C00148	HMDB00162
indoleacetate	protective	Amino Acid	Tryptophan Metabolism	C00954	HMDB00197
bilirubin (Z,Z)	protective	Cofactors and Vitamins	Hemoglobin and Porphyrin Metabolism	C00486	HMDB00054
X—23308	protective	/	/	/	/
1-arachidonoyl-GPC (20:4n6) *	protective	Lipid	Lysophospholipid	C05208	HMDB10395
benzoate	risk	Xenobiotics	Benzoate Metabolism	C00180	HMDB01870
ascorbate (Vitamin C)	risk	Cofactors and Vitamins	Ascorbate and Aldarate Metabolism	C00072	HMDB00044
2′-deoxyuridine	protective	Nucleotide	Pyrimidine Metabolism, Uracil containing	C00526	HMDB00012
pipecolate	protective	Amino Acid	Lysine Metabolism	C00408	HMDB00070
1-stearoyl-2-oleoyl-GPC (18:0/18:1)	protective	Lipid	Phosphatidylcholine (PC)	/	HMDB08038
X—18887	protective	/	/	/	/

* KEGG: Kyoto Encyclopedia of Genes and Genomes. https://www.kegg.jp, accessed on 20 September 2025; HMDB: Human Metabolome Database. https://hmdb.ca, accessed on 1 September 2025.

**Table 2:** Adenine-associated genes identified through SMR analysis.

Gene	Relevance to ASD	QTL Name	Top SNP ^*^	SNP Effect	*p_SMR_*	*p_SMR multi_*	*p_HEIDI_*
*ANP32E*		BrainMeta mQTL	rs7548543	risk	3.94 × 10^−4^	3.94 × 10^−4^	2.06 × 10^−1^
		BrainMeta sQTL	rs10788872	protective	2.14 × 10^−4^	4.44 × 10^−3^	5.92 × 10^−1^
*BAIAP2L1*	Iossifov et al. 2014 [75] Sanders et al. 2015 [76] Turner et al. 2017 [77] Satterstrom et al. 2020 [78]	BrainMeta mQTL	rs56997921	protective	4.36 × 10^−4^	4.36 × 10^−4^	2.13 × 10^−1^
*BNIP3*		BrainMeta mQTL	rs4550	risk	4.34 × 10^−4^	1.13 × 10^−3^	2.03 × 10^−1^
*CD52*		BrainMeta mQTL	rs12132137	risk	2.39 × 10^−4^	4.54 × 10^−3^	1.65 × 10^−1^
*COX7A1*		BrainMeta eQTL	rs1049339	risk	1.13 × 10^−4^	1.79 × 10^−3^	1.69 × 10^−1^
*FAM136A*		BrainMeta mQTL	rs13399467	protective	3.99 × 10^−4^	3.99 × 10^−4^	1.26 × 10^−1^
*FGGY*		BrainMeta mQTL	rs12080471	protective	3.20 × 10^−4^	2.57 × 10^−3^	3.64 × 10^−1^
*GINM1*		BrainMeta sQTL	rs9285521	protective	4.74 × 10^−4^	6.54 × 10^−4^	1.64 × 10^−1^
*GRM8*	Serajee et al. 2003 [79] Elia et al. 2012 [80] Prasad et al. 2012 [81] Zhou et al. 2022 [82]	BrainMeta mQTL	rs17866749	risk	4.67 × 10^−5^	4.67 × 10^−5^	5.30 × 10^−2^
*KATNA1*		BrainMeta mQTL	rs9766886	risk	4.08 × 10^−4^	4.08 × 10^−4^	1.10 × 10^−1^
*KATNAL2*	Sanders et al. 2012 [83] Stessman et al. 2017 [84] Kang et al. 2024 [85] Aspromonte et al. 2025 [86]	BrainMeta sQTL	rs72911209	risk	3.76 × 10^−4^	3.76 × 10^−4^	2.72 × 10^−1^
*LATS1*		BrainMeta sQTL	rs12175575	risk	2.59 × 10^−4^	2.59 × 10^−4^	5.53 × 10^−2^
		BrainMeta eQTL	rs9377228	protective	2.65 × 10^−4^	2.11 × 10^−3^	8.05 × 10^−1^
*LRP2BP*	Li et al. 2014 [87] Ruzzo et al. 2019 [88]	BrainMeta sQTL	rs7670667	risk	1.28 × 10^−4^	3.57 × 10^−4^	3.90 × 10^−1^
*LRP11*		BrainMeta sQTL	rs9322197	protective	3.03 × 10^−4^	3.03 × 10^−4^	6.16 × 10^−1^
*NEUROD6*		BrainMeta mQTL	rs2233402	risk	4.78 × 10^−4^	5.20 × 10^−3^	1.03 × 10^−1^
*NUP43*		BrainMeta sQTL	rs9688350	protective	2.90 × 10^−4^	2.90 × 10^−4^	5.65 × 10^−1^
*PGBD5*		BrainMeta mQTL	rs6541309	risk	4.74 × 10^−4^	2.12 × 10^−3^	7.86 × 10^−2^
*PIGX*		BrainMeta mQTL	rs12152276	risk	5.09 × 10^−5^	1.88 × 10^−4^	1.16 × 10^−1^
*RTEL1*		BrainMeta sQTL	rs41309367	protective	1.65 × 10^−4^	1.65 × 10^−4^	3.46 × 10^−1^

* Top SNP: SNP with the smallest *p_SMR_* value and highest significance.

**Table 3:** Proline-associated genes identified through SMR analysis.

Gene	Relevance to ASD	QTL Name	Top SNP ^*^	SNP Effect	*p_SMR_*	*p_SMR multi_*	*p_HEIDI_*
*ATP5MC2*		BrainMeta mQTL	rs3864905	risk	2.36 × 10^−4^	2.09 × 10^−3^	8.74 × 10^−2^
*AXIN1*		BrainMeta mQTL	rs12927169	risk	4.38 × 10^−4^	4.38 × 10^−4^	2.99 × 10^−1^
*ELMO1*		BrainMeta sQTL	rs10227527	protective	7.43 × 10^−5^	7.43 × 10^−5^	2.51 × 10^−1^
*F2RL3*		BrainMeta mQTL	rs773905	protective	3.51 × 10^−4^	2.59 × 10^−3^	4.23 × 10^−1^
*FBLN2*		BrainMeta mQTL	rs4684976	protective	4.89 × 10^−4^	4.89 × 10^−4^	2.65 × 10^−1^
*GPAM*		BrainMeta eQTL	rs117765391	risk	1.93 × 10^−4^	6.50 × 10^−4^	4.27 × 10^−1^
*GRM8*	Serajee et al. 2003 [79] Elia et al. 2012 [80] Prasad et al. 2012 [81] Zhou et al. 2022 [82]	BrainMeta mQTL	rs17862337	protective	4.71 × 10^−4^	4.71 × 10^−4^	5.06 × 10^−1^
*KTN1*		BrainMeta mQTL	rs10149408	risk	1.10 × 10^−4^	7.10 × 10^−4^	3.43 × 10^−1^
		BrainMeta sQTL	rs28681420	protective	6.96 × 10^−5^	7.28 × 10^−4^	6.06 × 10^−1^
*MBP*		BrainMeta mQTL	rs12958866	risk	1.90 × 10^−4^	1.66 × 10^−3^	8.16 × 10^−2^
*NME4*		BrainMeta sQTL	rs28694736	protective	1.92 × 10^−4^	1.54 × 10^−3^	6.29 × 10^−2^
*PIGN*		BrainMeta sQTL	rs17641789	protective	3.61 × 10^−4^	3.61 × 10^−4^	7.02 × 10^−1^
*SRL*		BrainMeta mQTL	rs4074565	protective	3.41 × 10^−4^	5.78 × 10^−3^	4.63 × 10^−1^
*STXBP6*		BrainMeta sQTL	rs912304	protective	1.37 × 10^−4^	8.00 × 10^−4^	2.04 × 10^−1^
*TECTB*		BrainMeta eQTL	rs11195869	risk	2.18 × 10^−4^	2.18 × 10^−4^	1.71 × 10^−1^
*TEX29*		BrainMeta mQTL	rs57211214	protective	1.09 × 10^−4^	6.60 × 10^−4^	3.13 × 10^−1^
*TMEM106B*		BrainMeta mQTL	rs11771383	protective	4.76 × 10^−4^	3.03 × 10^−3^	1.67 × 10^−1^
*ZC3H12D*		BrainMeta mQTL	rs409099	protective	3.61 × 10^−4^	1.89 × 10^−3^	2.14 × 10^−1^

* Top SNP: SNP with the smallest *p_SMR_* value and highest significance.

## Data Availability

The datasets analyzed during the current study are available from publicly accessible databases as follows: WADRC (ftp://ftp.biostat.wisc.edu/pub/lu_group/Projects/MWAS/, accessed on 1 September 2025), iPSYCH (https://ipsych.dk/fileadmin/ipsych.dk/Downloads/iPSYCH-PGC_ASD_Nov2017.gz, accessed on 1 September 2025), ADuLT (https://www.ebi.ac.uk/gwas/studies/GCST90275138, accessed on 1 September 2025), BrainMeta v2 cis-eQTL summary data (https://yanglab.westlake.edu.cn/data/SMR/BrainMeta_cis_eqtl_summary.tar.gz, accessed on 1 October 2025), BrainMeta v2 cis-sQTL summary data (https://yanglab.westlake.edu.cn/data/SMR/BrainMeta_cis_sqtl_summary.tar.gz, accessed on 1 October 2025), Brain-mMeta mQTL summary data (https://yanglab.westlake.edu.cn/data/SMR/Brain-mMeta.tar.gz, accessed on 1 October 2025). Other datasets used and/or analyzed during the current study are available from the corresponding author on reasonable request.

## Supplementary Materials

The following supporting information can be downloaded at https://www.mdpi.com/article/10.3390/genes17080874/s1, Figure S1: MR leave-one-out analysis results based on iPSYCH GWAS (exploration cohort). Figure S2: MR leave-one-out analysis results based on ADuLT GWAS (validation cohort). Figure S3: SMR EffectPlots comparing the effect sizes of SNPs in adenine and proline-GWAS (y-axis) with SNPs in BrainMeta-QTL data. (x-axis). Figure S4: The toxicological properties prediction results. Table S1: Metabolite information. Table S2: MR results via all methods (iPSYCH). Table S3: MR results via all methods (ADuLT). Table S4: Pleiotropy and heterogeneity test. Table S5: Leave-one-out analysis results (iPSYCH). Table S6: Leave-one-out analysis results (ADuLT). Table S7: SMR results of adenine. Table S8: SMR results of proline. Table S9: Gene-protein correspondence. Table S10: PPI data from STRING. Table S11: Protein-chemical interactions. Table S12: Toxicological properties prediction. Table S13: Molecular docking.

## Abbreviations

The following abbreviations are used in this manuscript:

ADME	Absorption, distribution, metabolism, and excretion
ASD	Autism spectrum disorder
B(a)P	Benzo(a)pyrene
BBB	Blood–brain barrier
CNS	Central nervous system
CSF	Cerebrospinal fluid
GO	Gene ontology
IVW	Inverse variance weighted
KEGG	Kyoto encyclopedia of genes and genomes
LD	Linkage disequilibrium
MR	Mendelian randomization
PPI	Protein–protein interaction
QTL	Quantitative trait locus
SMR	Summary-data-based mendelian randomization
WM	Weighted median
