# Supplementary material for: Multi-Omic Analysis of Cerebrospinal Fluid Metabolites in Autism Spectrum Disorder: Biomarker Identification, Metabolic Genetics Insights, and Network Toxicology
Source: Genes (Basel). 2026 Jul 27;17(8):874. doi: 10.3390/genes17080874 (PMC13512186; doi:10.3390/genes17080874)
Supplement: Supplementary file 1 [file genes-17-00874-s001.zip › Supplementary Figures_1-4.pdf]

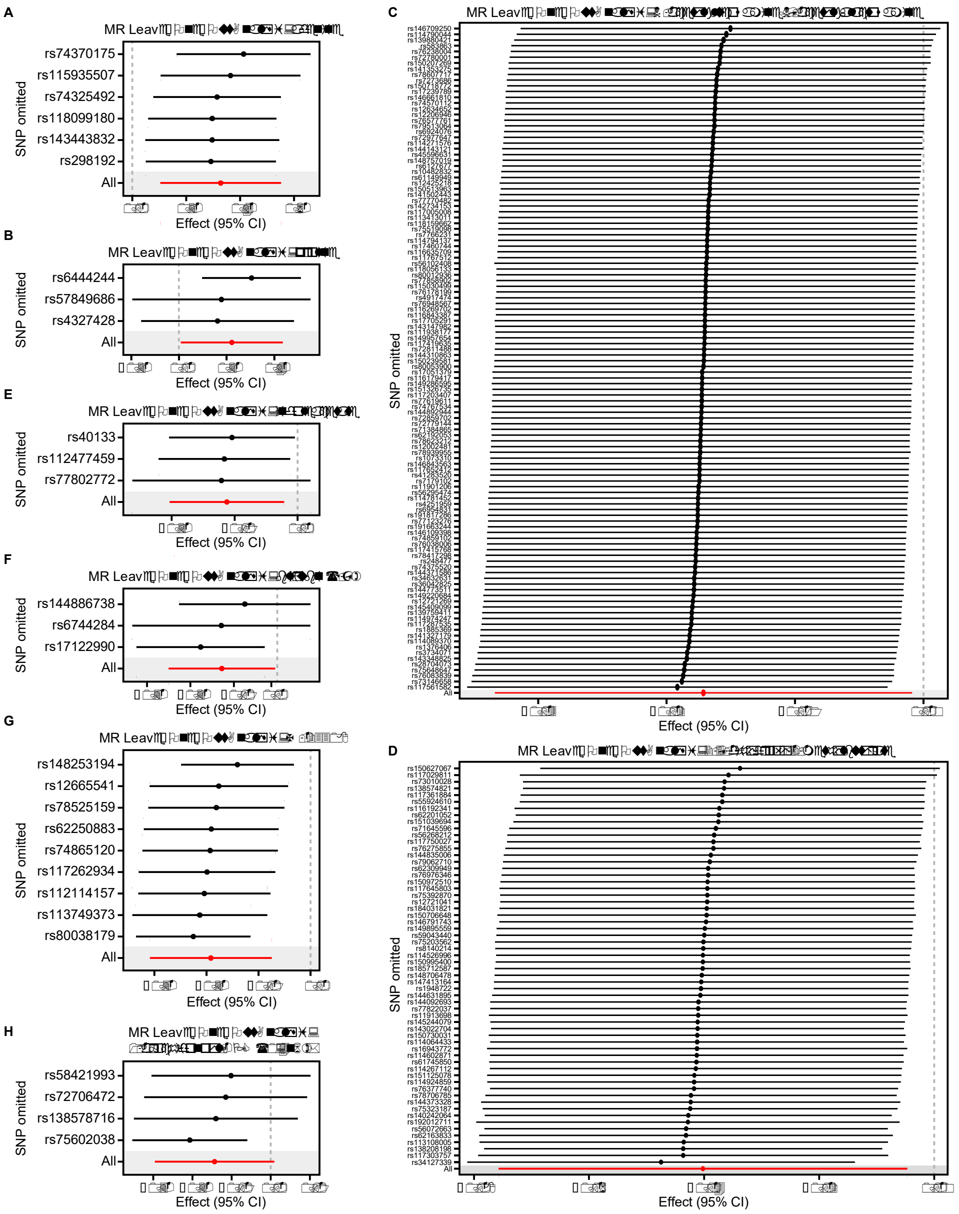

Supplementary Figure S1. MR leave-one-out analysis results based on iPSYCH GWAS (exploration cohort). (A) adenine; (B) proline; (C) N-acetylglucosamine/N-acetylgalactosamine; (D) 2,3-dihydroxy-2-methylbutyrate; (E) indoleacetate; (F) bilirubin (Z,Z); (G) X – 23308; (H) 1-arachidonoyl-GPC (20:4n6)*. In the leave-one-out plot, each SNP on the y-axis represents the MR analysis results after excluding that SNP, with the corresponding x-axis value being the effect size estimate. The overall effect size estimates for all metabolites change little after excluding a particular SNP, indicating that the contribution of this SNP to the overall MR effect is small and the overall effect is relatively stable.

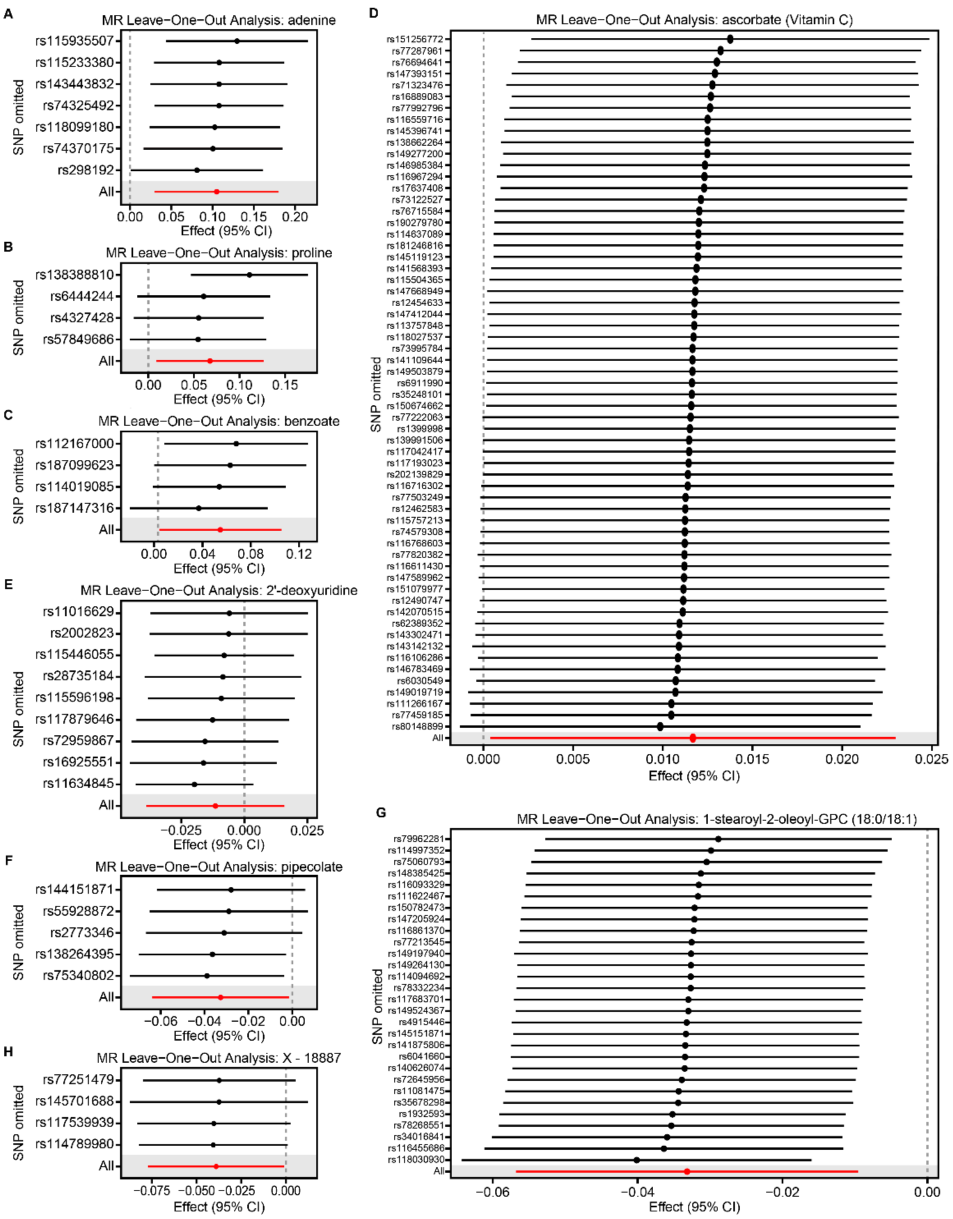

Supplementary Figure S2. MR leave-one-out analysis results based on ADuLT GWAS (validation cohort). (A) adenine; (B) proline; (C) benzoate; (D) ascorbate (Vitamin C); (E) 2'-deoxyuridine; (F) pipecolate; (G) 1-stearoyl-2-oleoyl-GPC (18:0/18:1); (H) X – 18887. In the leave-one-out plot, each SNP on the y-axis represents the MR analysis results after excluding that SNP, with the corresponding x-axis value being the effect size estimate. The overall effect size estimates for all metabolites change little after excluding a particular SNP, indicating that the contribution of this SNP to the overall MR effect is small and the overall effect is relatively stable.

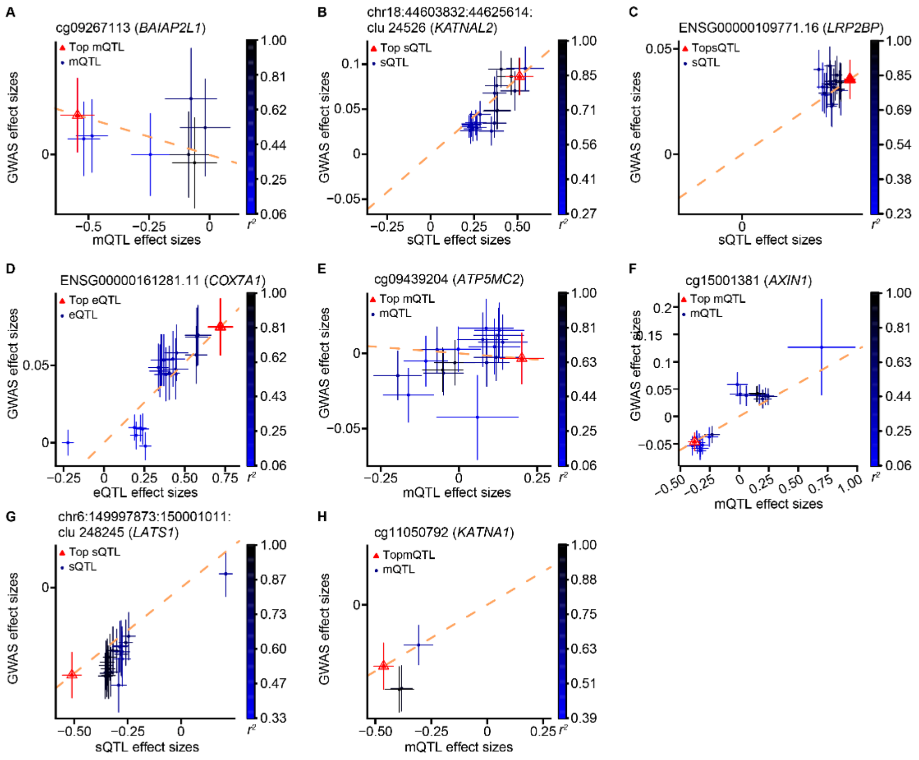

Supplementary Figure S3. SMR EffectPlots comparing the effect sizes of SNPs in adenine & proline-GWAS (y-axis) with SNPs in BrainMeta-QTL data. (x-axis). (A) adenine-*BAIAP2L1*; (B) adenine- *KATNAL2*; (C) adenine-*LRP2BP*; (D) adenine-*COX7A1*; (E) proline-*ATP5MC2*; (F) proline-*AXIN1*; (G) adenine-*LATS1*; (H) adenine-*KATNA1*.

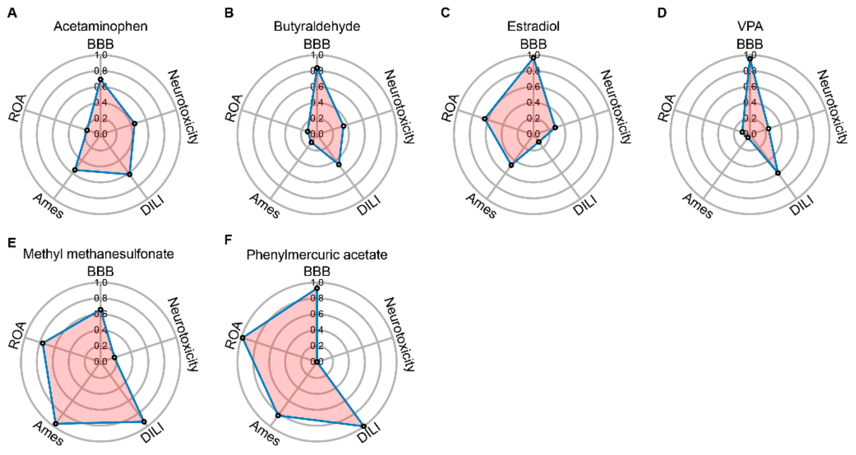

Supplementary Figure S4. The toxicological properties prediction results. (A) Acetaminophen; (B) Butyraldehyde; (C) Estradiol; (D) Valproic acid (VPA); (E) Methyl methanesulfonate; (F) Phenylmercuric acetate.
